# The effect of local anaesthetics on apoptosis and NETosis of human neutrophils in vitro: comparison between lidocaine and ropivacaine

**DOI:** 10.1007/s13577-023-00963-x

**Published:** 2023-08-17

**Authors:** Karolina Iwona Kulińska, Sandra Szałkowska, Mirosław Andrusiewicz, Małgorzata Kotwicka, Hanna Billert

**Affiliations:** 1 Chair and Department of Cell Biology, Poznan University of Medical Sciences, Rokietnicka 5D, 60-806 Poznań, Poland; 2 Chair of Anaesthesiology and Intensive Therapy, Poznan University of Medical Sciences, Przybyszewskiego 49, 60-355 Poznań, Poland

**Keywords:** Local anaesthetics, Apoptosis, NETosis, Neutrophils, Inflammation

## Abstract

There are immunological consequences to the method by which neutrophils undergo cell death. Neutrophil apoptosis, called silent death, leads to the resolution of inflammation, while NETosis deepens and prolongs the inflammatory response and is associated with a worse prognosis of severe infections, e.g., sepsis. Besides nociceptive inhibition, local anaesthetics modulate leukocyte functions, even at low, clinically relevant concentrations. There is currently no data on ropivacaine NETosis, and this study aimed to evaluate the impact of clinical concentrations of ropivacaine (0.0007, 0.007 and 1.4 mmol/L) and lidocaine (0.002, 0.02 and 4 mmol/L) on apoptosis and NETosis of adult peripheral blood neutrophils after 2 h of incubation. Neutrophil identification, apoptosis and NETosis were evaluated by flow cytometry using forward and side scatter characteristics and fluorescent labelling: CD15 for neutrophils identification; Annexin V and propidium iodide for apoptosis and citrullinated histone H3 and myeloperoxidase for NETosis. Lidocaine (4 mmol/L) and ropivacaine (1.4 mmol/L) induced early apoptosis in resting but not in stimulated neutrophils. Low doses of ropivacaine (0.0007 and 0.007 mmol/L) decreased the number of late apoptotic neutrophils, and the lowest dose slightly increased their viability. None of the drugs induced NETosis in resting neutrophils but decreased NETosis at clinical concentrations compared to PMA-stimulated 4 mM lidocaine, PMA-stimulated control, and 1.4 mM ropivacaine. The effect of lidocaine and ropivacaine on apoptosis and NETosis depended on neutrophil stimulation and drug concentrations. Ropivacaine tends to be cytoprotective at concentrations observed in plasma under local anaesthesia. Lidocaine enhanced NETosis at high concentration only in stimulated neutrophils. Thus, both drugs have the ability to change the course of inflammation.

**Supplementary Information:**

The online version contains supplementary material available at 10.1007/s13577-023-00963-x.

## Introduction

Neutrophils are the most abundant group of immune cells and are the first line of host defence against microorganisms. The most common mechanism by which they inactivate pathogens is respiratory burst and degranulation followed by phagocytosis inside the cell. In 2004, Brinkman et al. discovered a new mechanism called neutrophils extracellular traps (NET), which describes the way granulocytes entrap bacteria, viruses, and fungi in the extracellular space. NETs are formed from DNA decorated with nuclear content and granule proteins, e.g., myeloperoxidase (MPO), and neutrophil elastase (NE) released from the cell [1]. NET formation usually initiates the death of neutrophils called NETosis, a reactive oxygen species (ROS)-dependent process. It is induced extra-cellularly by protein kinase C (PKC) activators, e.g., phorbol 12-myristate 13-acetate (PMA). PMA-dependent NETosis occurs through the extracellular signal-regulated kinase (ERK) pathway (Raf–MEK–ERK pathway) and can be easily detected by fluorescent staining of MPO, NE or hyper-citrullinated histones, e.g., triple citrullinated histone three (CitH3), a marker of NETosis [2]. PMA is also a potent inducer of neutrophil apoptosis, leading to fast phosphatidylserine (PS) externalization with p38 mitogen-activated protein kinase (MAPK) stimulation [3].

Different immunological consequences occur depending on the type of neutrophil death [4]. Non-lytic deaths (apoptosis) generally exert an anti-inflammatory effect, while those leading to cell membrane damage (NETosis) worsen the inflammatory response, increase local tissue damage and induce chronic inflammation or autoimmunity [4]. Under physiological conditions, mature neutrophils die by spontaneous apoptosis, the cell remanent is recognized and phagocytosed by macrophages and is removed via the spleen and bone marrow [5, 6]. Apoptosis is crucial in rapidly removing granulocytes from the infected site, modulating the function of macrophages’ and resolving inflammation [7, 8]. For example, in septic patients, reduced neutrophil apoptosis was correlated with sepsis-induced acute respiratory distress syndrome (ARDS) [9]. During inflammation, the lifespan of granulocytes is prolonged, which is essential for the effective removal of pathogens and the inflammatory response [8]. NETosis predominates in both acute and chronic conditions and may affect the course of the disease [10]. Furthermore, NETs delay wound healing and inflammatory resolution [10]. Elevated NETs and the markers of NETosis, e.g., CitH3 have been found in patients with COVID-19, autoimmune diseases, sepsis and cancer [11–14]. Local anaesthetics (LAs) can alter neutrophil apoptosis and NETosis, thereby modulating the course of inflammation [15]. In Jurkat T-lymphoma cells, lidocaine at low, clinically relevant concentrations induced apoptosis via the mitochondrial pathway [16]. Furthermore, the current literature regarding the effect of LAs on neutrophil programmed cell death (PCD) during inflammation remains incomplete. However, the effect of LAs probably depends on the type and functional state of the neutrophils and the stage of inflammation. Chiang et al. suggest that lidocaine delays the apoptosis of activated neutrophils and inhibits their phagocytosis by macrophages, thus modulating the resolution of acute inflammation [17]. Moreover, a recent study showed that lidocaine and bupivacaine can inhibit NET formation [15]. Ropivacaine, a commonly used LA, is less neurotoxic and cardiotoxic than lidocaine and shows a stronger pharmacological effect. According to the current literature, ropivacaine at clinically relevant concentrations increases neutrophil NO production and decreases adhesion, chemotaxis and migration with no effect on neutrophil apoptosis [18–21]. Currently, there is a lack of reports on the role of ropivacaine in NET formation and NETosis. The running hypothesis is that ropivacaine modulates rested and activated neutrophil NETosis, and the effect depends on the drug concentration. This work evaluated the apoptosis and NETosis of resting and PMA-stimulated neutrophils and compared the impact of clinically relevant concentrations of ropivacaine and lidocaine on the two types of neutrophil death.

## Materials and methods

The experiments were conducted according to the Declaration of Helsinki after receiving local Bioethical Commission approval (702/19, Poznan University of Medical Sciences). Every volunteer signed a written informed consent form. Venous blood (18 mL) was collected under sterile conditions in Monovette EDTA-containing Vacutainers (Sarstedt, Nümbrecht, Germany) from healthy, non-smoking men aged 20–40 (n = 9). The experiments began within an hour of the blood collection.

## Polymorphonuclear cells isolation

Polymorphonuclear cells (PMN) were isolated by density gradient centrifugation using Gradisol G (density = 1,119 g/cm^3^; Aqua-Med, Łódź, Poland). Blood (4.5 mL) was layered onto the Gradisol G surface and centrifuged at 400 rcf for 30 min at room temperature (RT) (Centrifuge 5804R, Eppendorf, Germany). The plasma and peripheral blood mononuclear cells (PBMC) were removed, and the buffy coat of PMNs was collected into a 50 mL tube, washed twice with PBS (w/o Ca^2+^/Mg^2+^) and cells were counted under a Jenaval Carl Zeiss microscope (Carl Zeiss, Jena, Germany) using a Bürker chamber. PMNs at a concentration of 4 × 10^6^ cells/mL were resuspended in Hank’s balanced salt solution without Ca^2+^/Mg^2+^ (HBSS) obtained from the Institute of Immunology and Experimental Therapy, Polish Academy of Sciences (Wrocław, Poland). The viability and purity of the PMNs were over 97% and 95%, respectively, according to the 0.4% Trypan blue dye exclusion (Sigma Aldrich, St. Louis, MO, USA), Türk’s solution staining (Aqua-Med) and flow cytometry forward (FSC) and side scatter (SSC) characteristics.

## Flow cytometry neutrophils identification

Identification of neutrophils was based on the phycoerythrin (PE)-conjugated CD15 antibody staining [22]. In brief, following density gradient centrifugation, the buffy coat of PMNs was resuspended in a staining buffer (PBS supplemented with 0.5% BSA and 2 mM EDTA) to obtain 4 × 10^3^ cells/µL. 100 µL of cell suspension was incubated with PE-conjugated mouse anti-human CD15 antibody (555402; BD Biosciences, UK) at 4 °C for 30 min in the dark. Next, cells were washed twice with staining buffer and analysed by flow cytometry using a FACS Canto II (BD, San Jose, USA) and FACS Diva software. The whole procedure, from cell staining to the final result, took less than 1 h. Ten thousand events were collected under unchanged cytometer parameters: voltages for photomultipliers, collection parameters, and compensation. Before each experiment, a calibration test was performed using CS&T beads (Cytometer Setup & Tracking Beads Kit, BD).

## Pre-incubation with lidocaine and ropivacaine

Neutrophils were seeded onto a 12-well plate and incubated with equipotent concentrations of lidocaine (0.002 mmol/L, 0.02 mmol/L, and 4 mmol/L) and ropivacaine (0.0007 mmol/L; 0.007 mmol/L; 1.4 mmol/L). Ropivacaine is approximately 3 times more potent than lidocaine. The concentrations were set based on previous literature and corresponded to those observed in tissues and plasma during LA and pain management, e.g., the highest concentrations were observed in tissues near the spinal cord after subarachnoid block [23–26]. Cells incubated with PBS were set as control. Incubation was conducted under standard conditions at 37 °C, 5% CO_2_ (Hera Cell 150; Thermo Scientific, MA, USA) for 2 h.

## PMA-stimulated apoptosis and NETosis

After the LA pre-treatment, cells were transferred into cytometric tubes and incubated with 600 ng/mL PMA (stimulated neutrophils) or PBS (resting neutrophils) for 15 min (37 °C, 5% CO_2_, 98% humidity). Next, cells were washed with PBS to stop the reaction and centrifuged at 300 rcf at 15 °C for 10 min. Based on previous experiments, the PMA concentrations and stimulation times were optimized [27–30].

## Apoptosis assay using Annexin V and propidium iodide

Apoptosis was assessed and compared between resting and PMA-stimulated neutrophils pre-exposed to LAs or PBS. After washing in PBS, neutrophils were resuspended in 1 × binding buffer (556547; BD Biosciences Pharmingen, San Diego, CA, USA) and incubated with 4 µL fluorescein (FITC)-conjugated Annexin V and 2.5 µL propidium iodide (PI) (BD Biosciences) at RT in the dark for 15 min. Neutrophils were analysed within 1 h by flow cytometry using FACS Diva software v.6 (BD Biosciences). At least 10,000 events were evaluated according to the gating strategy. Viable cells showed no fluorescence of Annexin V-FITC or PI. Early apoptotic neutrophils were positive for Annexin V-FITC but negative for PI; late apoptotic cells were positive for both Annexin V-FITC and PI, while necrotic cells were only PI-positive. Apoptotic neutrophils were determined as the percentage of Annexin V-positive cells.

## Identification of NETosis

H3 citrullination at R2, R8 and R17 residues, and the presence of MPO between the chromatin fibres were chosen as the markers of NETosis [2]. Rested and PMA-stimulated neutrophils preincubated with LAs or PBS were resuspended in staining buffer and incubated with primary rabbit polyclonal anti-histone H3 (Citrulline R2 + R8 + R17) antibody (1:400; ab5103; Abcam, Great Britain) at RT in the dark for 30 min. Next, neutrophils were washed and incubated with a secondary goat anti-rabbit Alexa Fluor 647-conjugated IgG antibody (1:200; A-21244; Thermo Fisher Scientific, Waltham, MA, USA) and FITC-conjugated mouse anti-human myeloperoxidase (1:50; ED7261; Exbio, Czech Republic) at RT in the dark for 30 min. Cells were washed and analysed by flow cytometry within 1 h using a FACS Canto II and FACS Diva Software as above. The percent of MPO FITC ^high^ and CitH3 Alexa Fluor 647^high^ neutrophils were determined to be NET-positive.

## Statistics

Statistical analysis was performed using Graph Pad Prism v.6 (GraphPad Software, Inc., La Jolla, CA, USA), and the artwork graphical presentation was adjusted in Corel Draw software 2018. Normally distributed data were analysed using the Shapiro–Wilk test, and the equality of variances was assessed with a Fisher`s *F* test. In the case of parametric distribution with equal variances unpaired *t* test was used. In the case of unequal variances, differences between the two groups were analysed with an unpaired *t* test with Welch’s correction. In the case of nonparametric distribution, a Mann–Whitney *U* test was used. The Friedmann test with *post hoc* Dunn’s test was performed for more than two groups to compare differences between concentrations of one drug, and a Kruskal–Wallis test with Dunn’s post hoc test was used to compare stimulation within concentrations and drug differences. Results were presented as a median with an interquartile range, and a *p* value of < 0.05 was considered statistically significant.

## Results

### Neutrophils identification

**Fig. 1:**
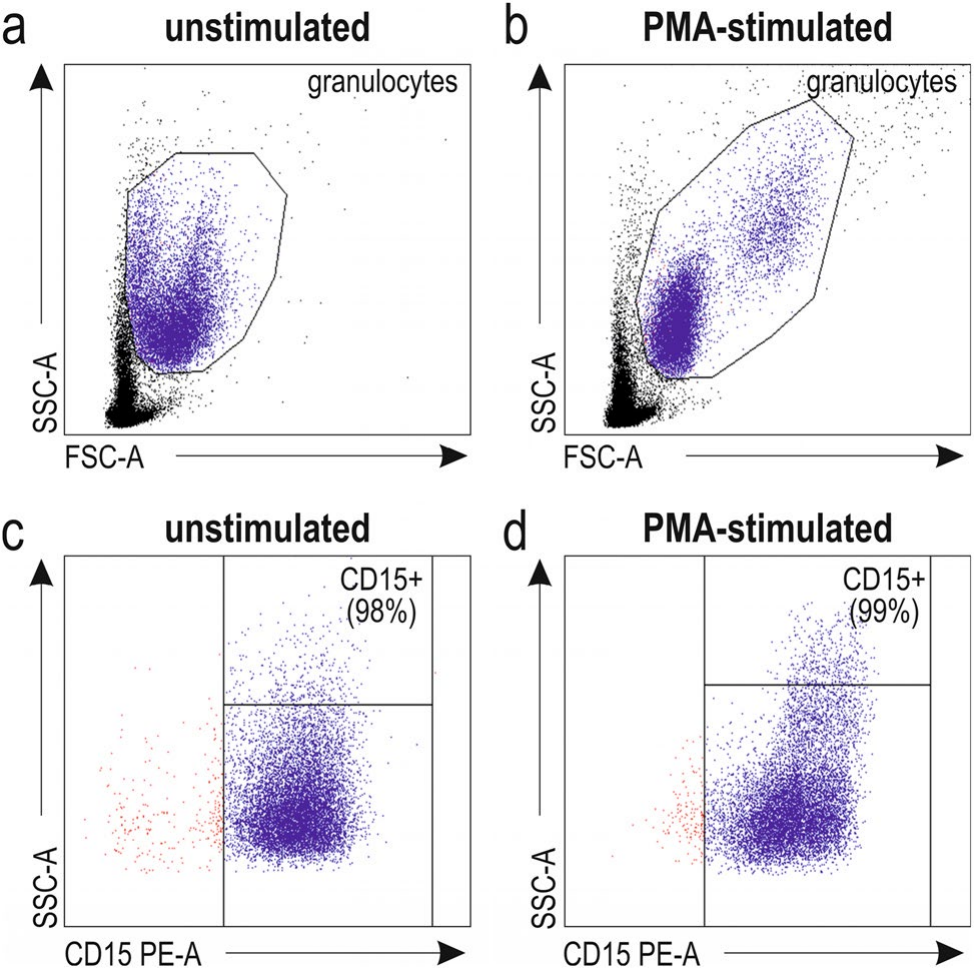
Identification of neutrophils. FSC and SSC characteristics (**a**, **b**) and CD15+ PE staining (indicated as purple dots) of resting and PMA-stimulated granulocytes (**c**, **d**). Representative dot plots. *FSC-A* forward scatter characteristics-area; *SSC-A* side scatter characteristics-area; *PE-A* phycoerythrin-area; *PMA* phorbol 12-myristate 13-acetate

The cytometric analysis was performed to identify the good-quality samples (granulocytes > 95%). An example of cytometric identification of neutrophils is shown in Fig. 1. Debris and lymphocytes were excluded from the analysis based on FSC/SSC characteristics (Fig. 1a, b). Granulocytes were gated as FSC^high^ and SSC^high^. Only those cells with high expression of CD15 antigen (> 98%) were used for subsequent experiments (Fig. 1c, d).

**Fig. 2:**
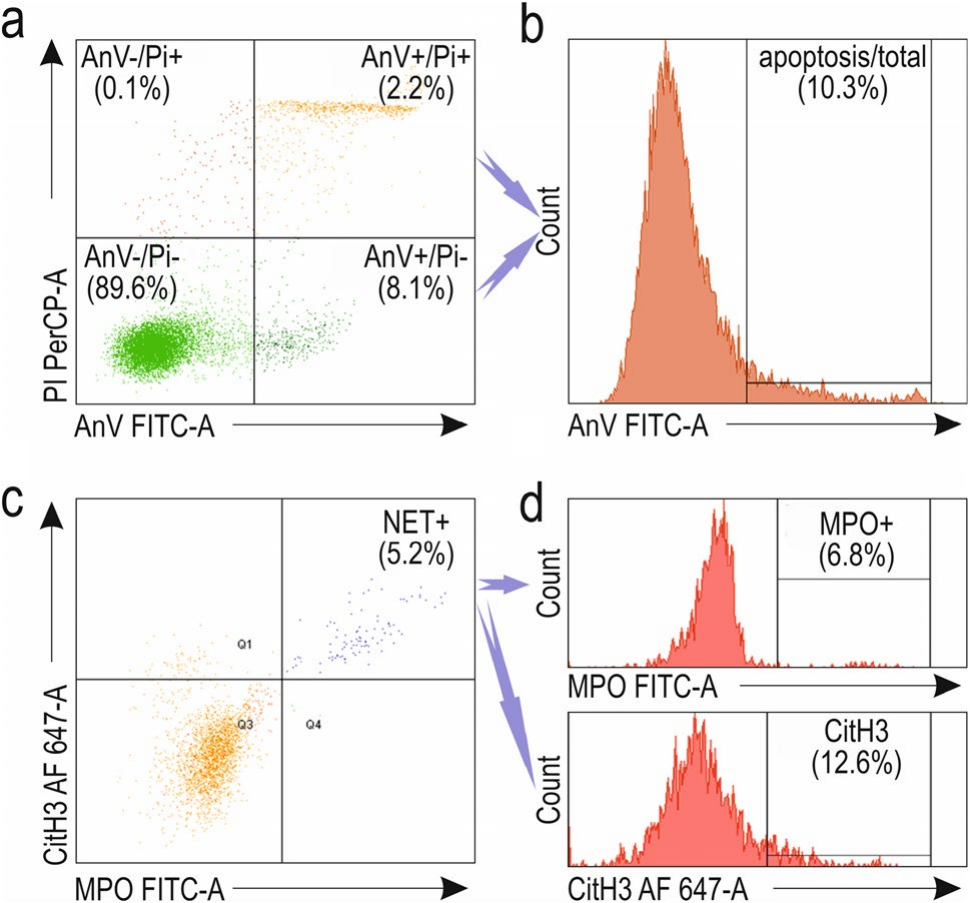
Identification and evaluation of PMA-induced apoptosis and NETosis of human neutrophils. Viable (Annexin V^ –^ /PI^ – ^), early apoptotic (Annexin V^+^ /PI^–^ ), late apoptotic (Annexin V^+^ /PI^+^) and necrotic (Annexin V^–^ /PI^+^) neutrophils (**a**). Apoptosis (early and late) gated as AnnexinV^high^ (**b**). NETosis gated as MPO^high^ and CitH3^high^ (**c**, **d**). *FITC-A* fluorescein isothiocyanate-area; *AF 647-A* Alexa Fluor 647-area; *PerCP-A* Peridinin chlorophyll protein-area; *AnV* Annexin V; *CitH3* R2, R8, R17 citrullinated histone H3; *MPO* myeloperoxidase; *PI* propidium iodide; *PMA* phorbol 12-myristate 13-acetate

Apoptosis was assessed based on Annexin V/PI staining. Viable neutrophils with > 98% CD15 expression were represented as negative for Annexin V FITC or PI. Early apoptotic granulocytes were evaluated as the cells positive for Annexin V FITC only; late apoptotic cells showed high fluorescence of Annexin V FITC and PI, while necrotic neutrophils were those positive only for PI (Fig. 2a). Cell quantities were expressed as percentages. Apoptosis was identified as Annexin V FITC fluorescent neutrophil percentages (Fig. 2b). NETosis was evaluated based on MPO and CitH3 staining. Only granulocytes showing fluorescence of both antigens were considered NETotic and expressed as % of MPO FITC^+^/CitH3 Alexa Fluor 647^+^ cells (Fig. 2c, d).

## PMA-stimulated apoptosis and NETosis of neutrophils

**Fig. 3:**
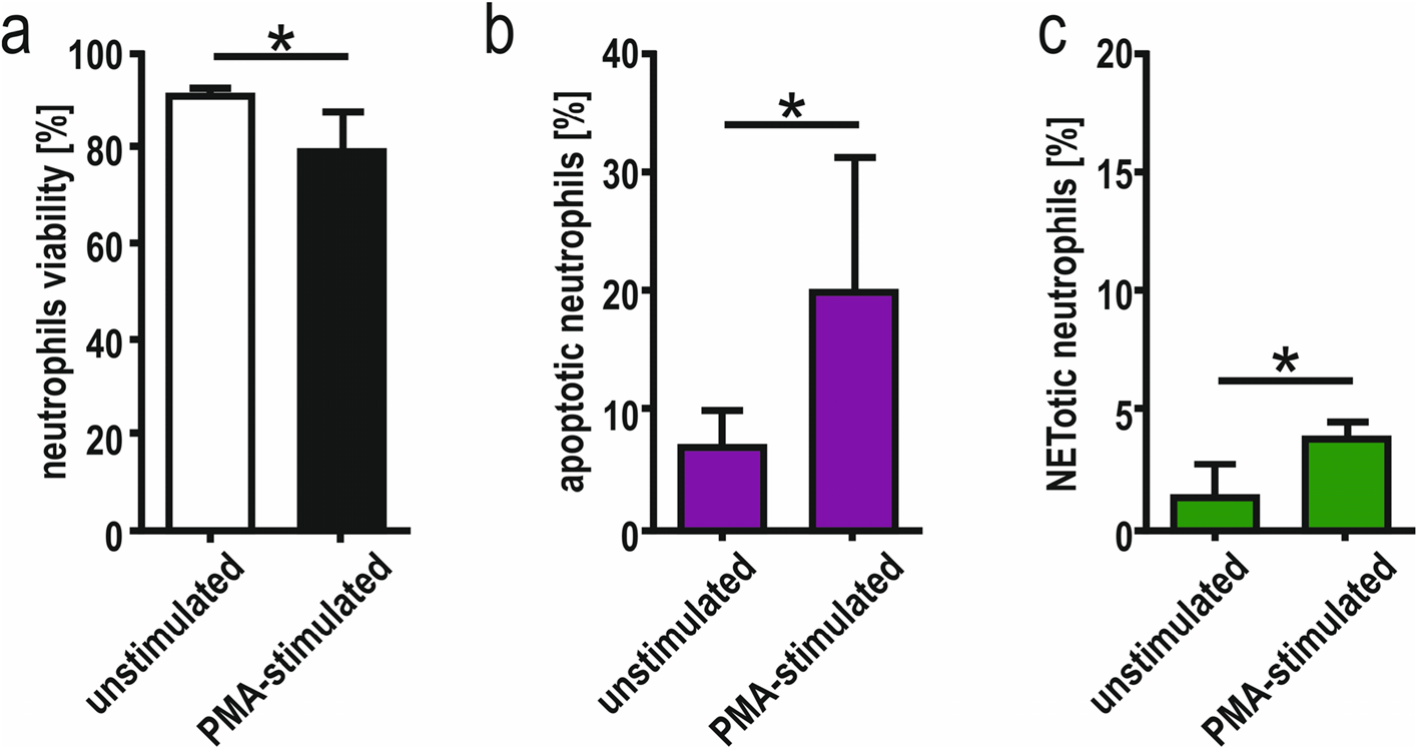
Quantitative evaluation of viability (**a**), apoptosis (**b**) and NETosis (**c**) after 15 min stimulation with 600 ng/mL PMA. Data are median with the interquartile range; *us* unstimulated neutrophils; *PMA* phorbol 12-myristate 13-acetate (600 ng/mL); *p < 0.05

Neutrophils were treated with 600 ng/mL PMA for 15 min to analyse the PMA-stimulated viability, apoptosis and NETosis. In the case of the PMA-treated samples compared to the unstimulated control, we observed decreased viability (80% vs 92%; *p* = 0.0252, Fig. 3a), three times increased apoptosis (20% vs 7.1%; *p* = 0.0174; Fig. 3b) and twice NETosis (4% vs 1.4%; *p* = 0.0281; Fig. 3c).

## Lidocaine and ropivacaine pre-incubation effect on resting and PMA-stimulated neutrophils

### Apoptosis

**Fig. 4:**
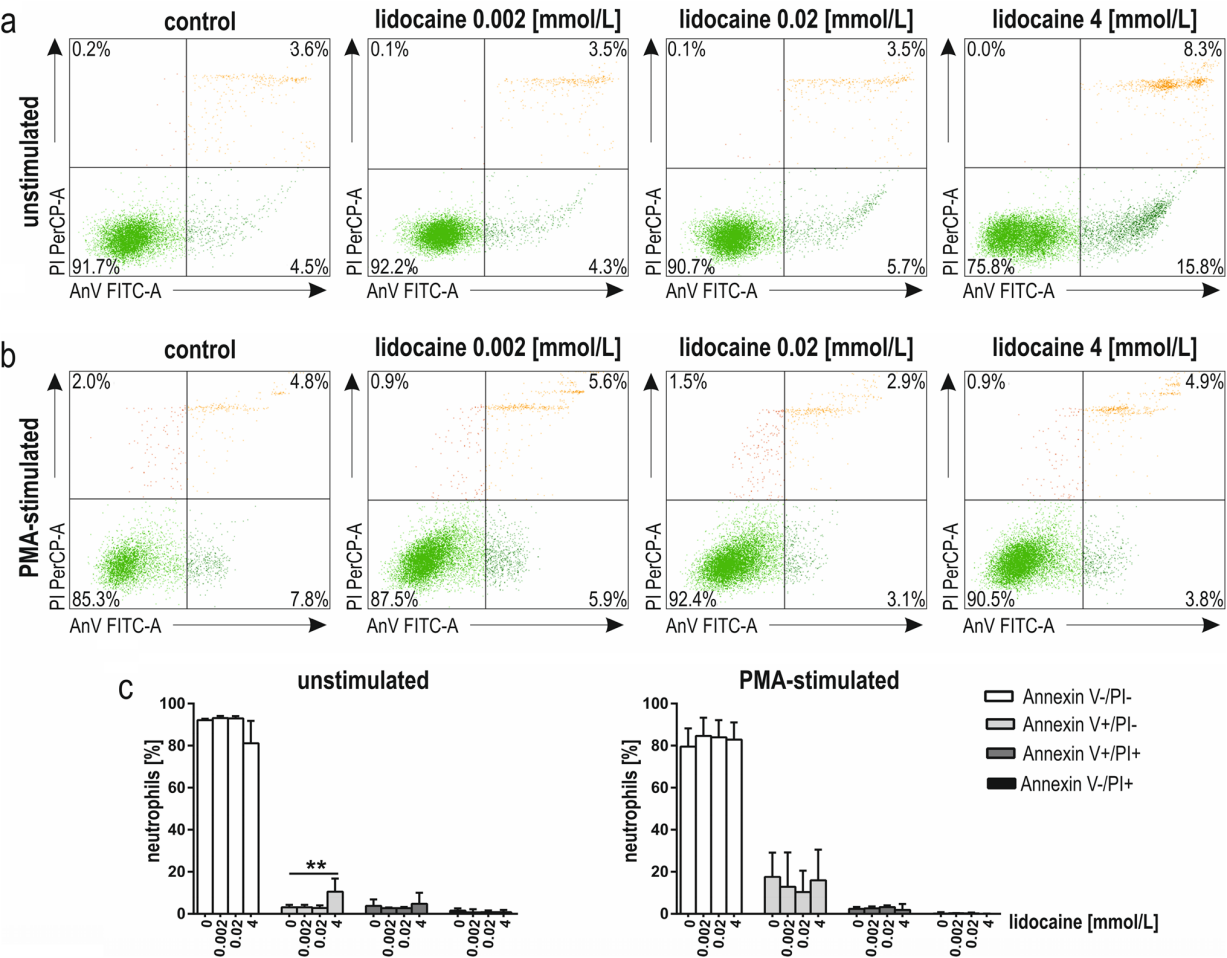
Apoptosis of human neutrophils pre-incubated for 2 h with lidocaine (0.002 mmol/L, 0.02 mmol/L, 4 mmol/L). Representative dot plots from a single experiment, where Annexin V FITC and PI staining was used (**a**, **b**); viable cells—lower left quadrant (Annexin V FITC^–^/PI^–^), early apoptotic cells—lower right quadrant (Annexin V FITC^+^ /PI^–^ ), late apoptotic cells—upper right quadrant (Annexin V FITC^+^/PI^+^), necrotic cells—upper left quadrant (Annexin V FITC^–^ /PI^+^) of resting (**a**) and PMA-stimulated neutrophils (**b**); Quantitative representation of the effect of lidocaine on the viability and apoptosis of resting and PMA-stimulated neutrophils (**c**). Results are median with the interquartile range from eight independent experiments. **p < 0.01; *FITC-A* fluorescein isothiocyanate-area; *PerCP-A* Peridinin chlorophyll protein-area; *AnV* Annexin V; *PI* propidium iodide; *PMA* phorbol 12-myristate 13-acetate

Analysing the unstimulated and PMA-stimulated cells (Fig. 4a, b), a pre-incubation of 2 h with lidocaine induced early stages of apoptosis (neutrophils positive for Annexin V only) in resting but not in PMA-stimulated neutrophils. The effect was observed at 4 mmol/L lidocaine (*p* < 0.01; Fig. 4c). In other lidocaine concentrations, the observed effects were not significant (*p* > 0.05; Fig. 4c). The further raw data are presented in Supplementary Table S1.

**Fig. 5:**
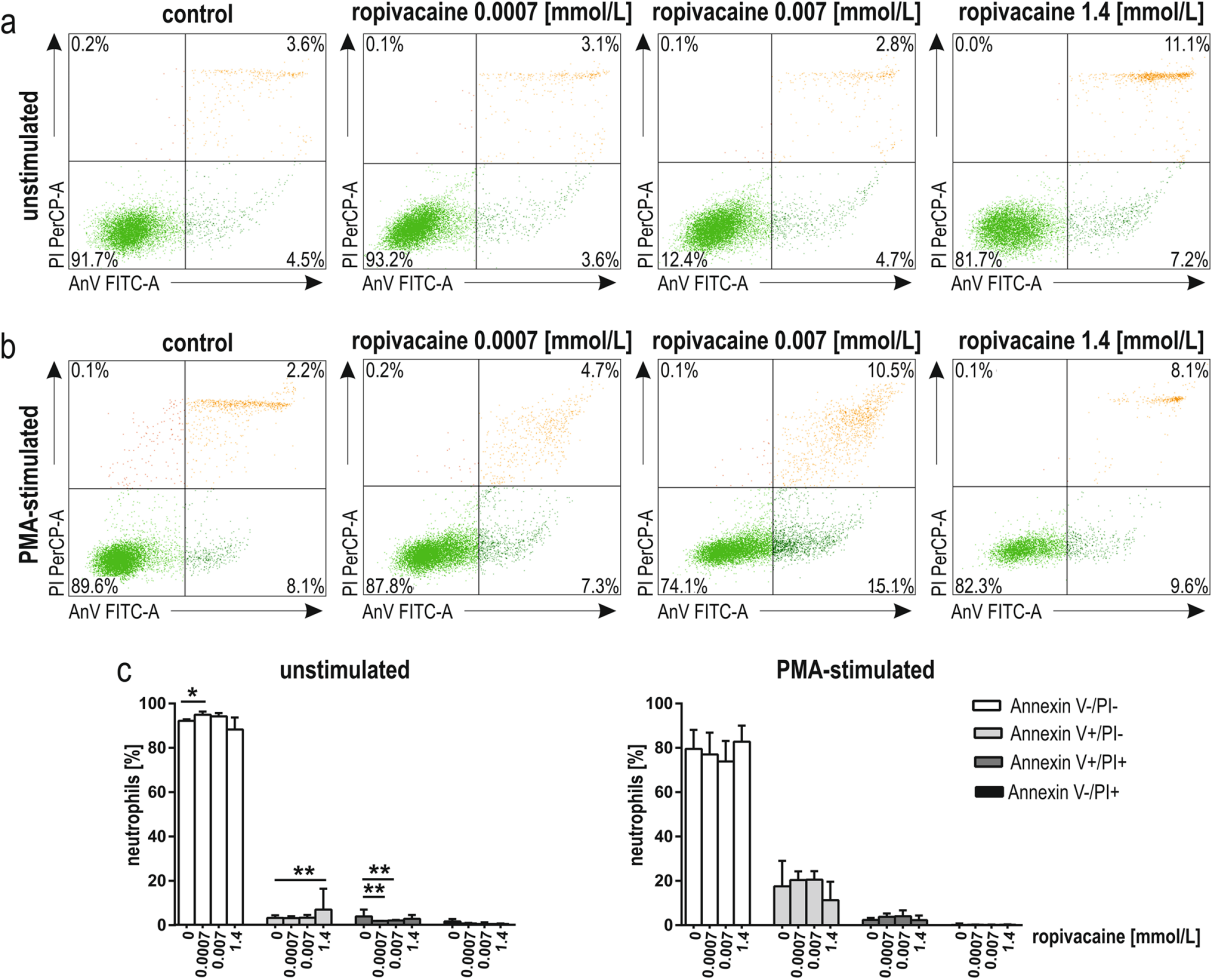
Apoptosis of human neutrophils pre-incubated for 2 h with ropivacaine (0.0007 mmol/L, 0.007 mmol/L, 1.4 mmol/L). Representative dot plots from a single experiment, where Annexin V FITC and PI staining was used (**a**, **b**); viable cells—lower left quadrant (Annexin V FITC^–^/PI^–^), early apoptotic cells—lower right quadrant (Annexin V FITC^+^ /PI^–^ ), late apoptotic cells—upper right quadrant (Annexin V FITC^+^/PI^+^), necrotic cells—upper left quadrant (Annexin V FITC^–^ /PI^+^) of resting (**a**) and PMA-stimulated neutrophils (**b**); Quantitative representation of the effect of ropivacaine on the viability and apoptosis of resting and PMA-stimulated neutrophils (**c**). Results are median with the interquartile range from eight independent experiments. *p < 0.05, **p < 0.01. *FITC-A* fluorescein isothiocyanate-area; *PerCP-A* Peridinin chlorophyll protein-area; *AnV* Annexin V; *PI* propidium iodide; *PMA* phorbol 12-myristate 13-acetate

A pre-incubation of 2 h with ropivacaine induced early stages of apoptosis (neutrophils positive for Annexin V only) in resting but not in PMA-stimulated neutrophils (Fig. 5a, b). The effect was observed at 1.4 mmol/L ropivacaine (*p* < 0.01, Fig. 5c). Clinically relevant concentrations of ropivacaine, namely, 0.0007 mmol/L and 0.007 mmol/L decreased the fraction of late apoptotic cells (positive for Annexin V and PI; *p* < 0.01; Fig. 5c). The viability of resting neutrophils preincubated with 0.0007 mmol/L ropivacaine was slightly increased compared to the control (*p* < 0.05; Fig. 5c). The further raw data are presented in Supplementary Table S1.

Comparing lidocaine and ropivacaine, the rates of early apoptotic cells were similar for both drugs (*p* > 0.05).

### NETosis

**Fig. 6:**
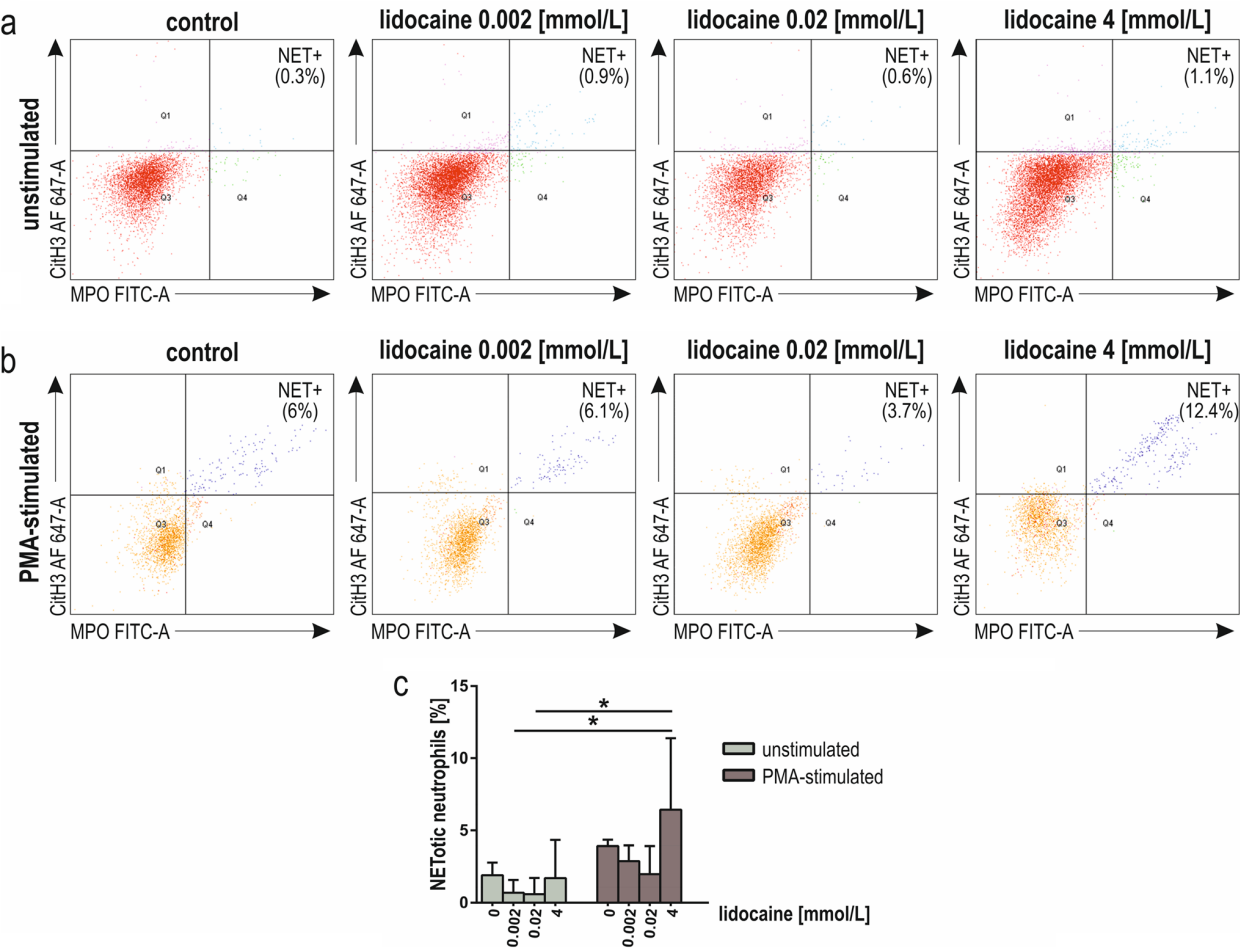
NETosis of human neutrophils pre-incubated for 2 h with lidocaine (0.002 mmol/L, 0.02 mmol/L, 4 mmol/L). Representative dot plots from a single experiment, where MPO FITC and CitH3 Alexa Fluor 647 staining was used; NET +ET^+^cells—upper right quadrant (MPO FITC^+^ /CitH3 Alexa Fluor 647^+^) in resting (**a**) and PMA-stimulated neutrophils (**b**). Quantitative representation (percentage of NET + cells) of the effect of lidocaine on the NETosis of resting and PMA-stimulated neutrophils (**c**). Results are median with the interquartile range from nine independent experiments. *FITC-A* fluorescein isothiocyanate-area; *AF 647-A* Alexa Fluor 647-area; *CitH3* R2, R8, R17 citrullinated histone H3; *MPO* myeloperoxidase; *PMA* phorbol 12-myristate 13-acetate; *p < 0.05

NETosis in unstimulated and PMA-stimulated neutrophils, pre-incubated for 2 h with lidocaine, were analyzed by flow cytometry (Fig. 6a, b). The 4 mmol/L lidocaine increased NETosis of PMA-stimulated neutrophils compared to control. Still, the effect was not statistically significant (Fig. 6c). Interestingly, we observed increased PMA-induced NETosis for the highest lidocaine concentration compared to unstimulated the lowest clinically relevant concentrations of the drug (*p* < 0.05, Fig. 6c).

**Fig. 7:**
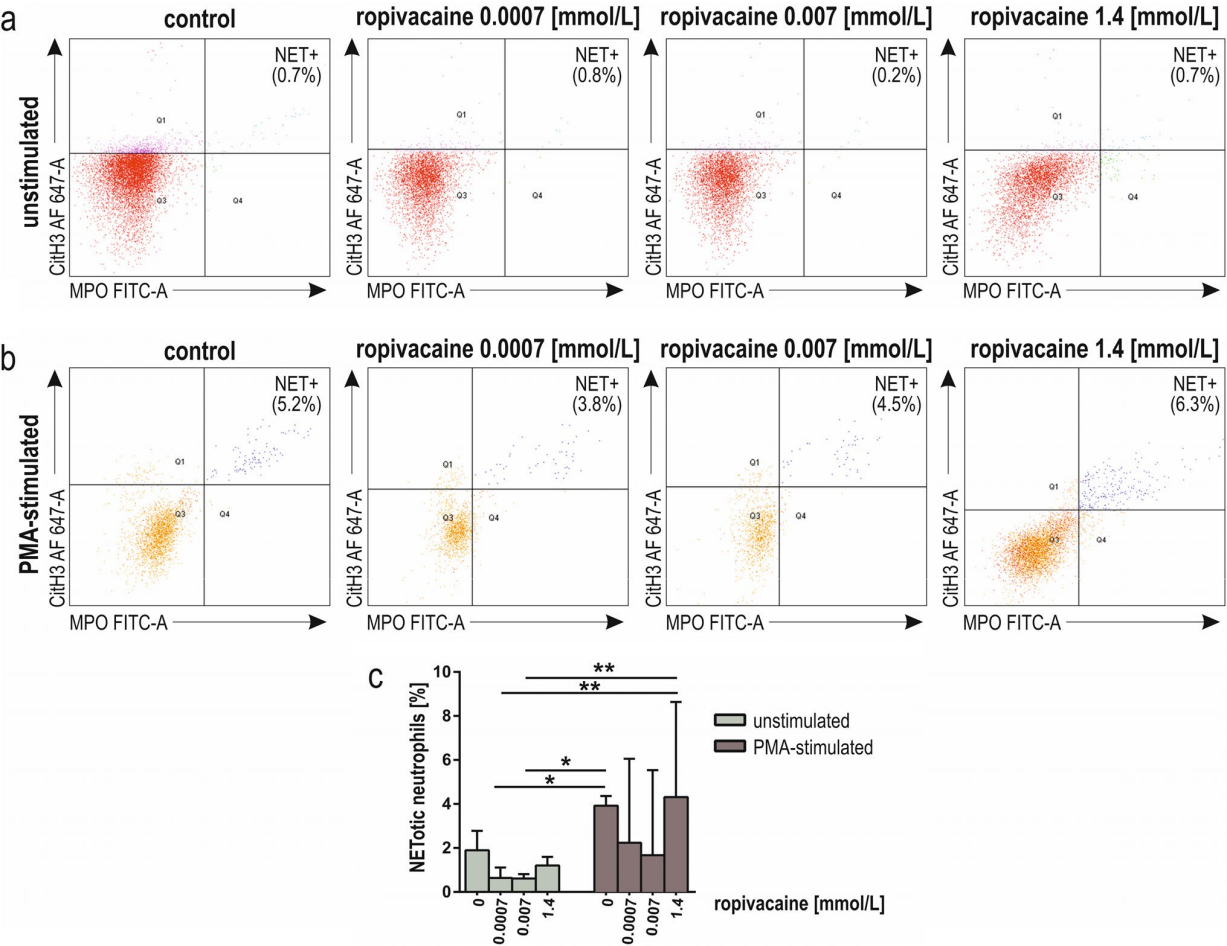
NETosis of human neutrophils pre-incubated for 2 h with ropivacaine (0.0007 mmol/L, 0.007 mmol/L, 1.4 mmol/L). Representative dot plots from a single experiment, where MPO FITC and CitH3 AF 647 staining was used; NET + cells—upper right quadrant (MPO FITC^+^ /CitH3 AF 647 ^+^) in resting (**a**) and PMA-stimulated neutrophils (**b**). Quantitative representation (percentage of NET + cells) of the effect of lidocaine on the NETosis of resting and PMA-stimulated neutrophils (**c**). Results are median with the interquartile range from nine independent experiments. *p < 0.05, **p < 0.01. *FITC-A*—fluorescein isothiocyanate-area; *AF 647-A* Alexa Fluor 647-area; *CitH3*—R2, R8, R17 citrullinated histone H3; *MPO*—myeloperoxidase; *PMA* phorbol 12-myristate 13-acetate

NETosis in unstimulated and PMA-stimulated neutrophils, pre-incubated for 2 h with ropivacaine, were analyzed by flow cytometry (Fig. 7a, b). We observed significantly lower NETosis in resting neutrophils preincubated with the clinically relevant drug concentration compared to both PMA-stimulated control (*p* < 0.05) and 1.4 mmol/L ropivacaine (*p* < 0.01; Fig. 7c).

**Fig. 8:**
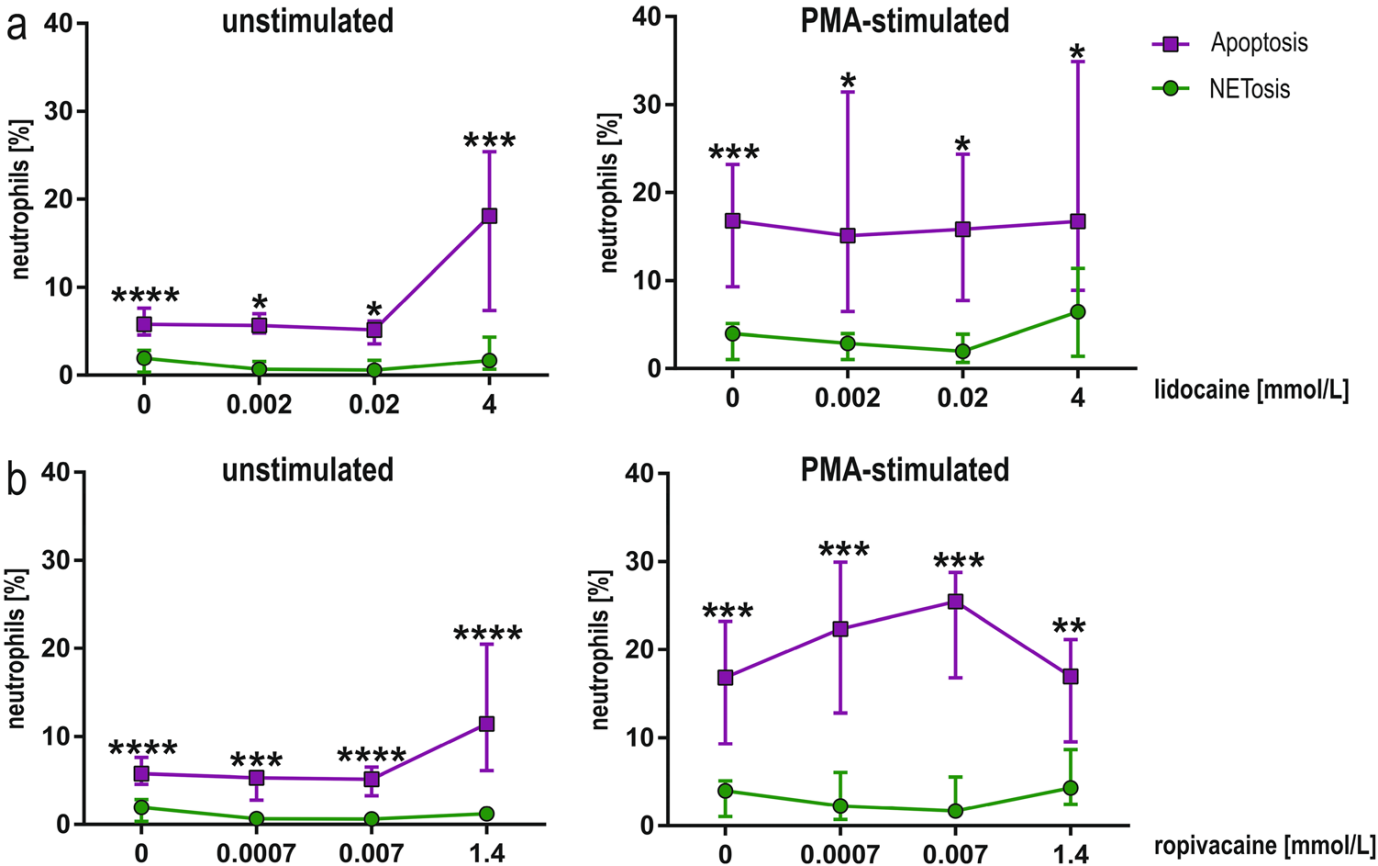
Apoptosis and NETosis of resting and PMA-stimulated human neutrophils pre-exposed to lidocaine (**a**) and ropivacaine (**b**). Data are median with the interquartile range; % apoptotic neutrophils are Annexin V-positive cells; *p < 0.05, **p < 0.01, ***p < 0.001, ****p < 0.0001; *PMA* phorbol 12-myristate 13-acetate

None of the drugs affected NETosis in resting neutrophils (Figs. 6 and 7);

Considering the apoptosis and NETosis in resting and PMA-stimulated neutrophils (independently of the drug used), the apoptosis was observed in a higher percentage compared to NETosis (Fig. 8a, b and Supplementary Table S2). The differences were stronger in the case of ropivacaine than lidocaine.

## Discussion

Herein, we observed that lidocaine and ropivacaine at clinically relevant concentrations induced apoptosis in resting but not in PMA-stimulated neutrophils. The effect was observed after 2 h of incubation at millimolar concentrations of the drugs. Ropivacaine at low doses, similar to those observed in plasma under LA, reduced the number of late apoptotic neutrophils and increased their viability (0.0007 mmol/L). Lidocaine (4 mmol/L) increased NETotic neutrophils simulated with PMA (compared to the unstimulated lowest doses), while neither drug-induced NETosis in resting neutrophils. Ropivacaine did not change the NETosis per se. Still, interestingly, we found lower NETosis process in resting neutrophils pre-treated with clinically relevant concentrations of the drug compared to PMA-stimulated control and the highest, 1.4 mmol/L dose of the drug. To the best of our knowledge, this is the first study to compare the effect of lidocaine and ropivacaine on neutrophil NETosis in vitro*,* and we found no differences between both anaesthetics.

While commonly used for nociceptive inhibition, lidocaine and ropivacaine also alter the function of macrophages, granulocytes, mast cells, and lymphocytes, which may impact wound healing, blood coagulation and clotting, development and suppression of the inflammatory response, and the course of infections [31, 32]. According to the available literature, LAs increase apoptosis of granulocytes and lymphocytes, which may be associated with postoperative complications, such as the increased risk of developing sepsis, multiorgan failure and tumour recurrence [31, 32]. Recent findings indicate that lidocaine can inhibit NETosis, thus preventing cancer recurrence and reducing the severity of both COVID and sepsis [33].

## PMA-induced apoptosis and NETosis of human neutrophils

Our study reported approximately 6% apoptotic and 2% NETotic resting neutrophils. Neutrophils are constitutively programmed to undergo apoptosis, a process called silent death. Ageing, fully segmented neutrophils show characteristics of apoptotic cells, including cell shrinkage, chromatin condensation and DNA fragmentation. According to Brinkmann et al., unstimulated neutrophils demonstrate NETosis of below 5% [34]. The potent PKC activator PMA inhibits apoptosis in many cell types, e.g., cancer cells [35, 36]. However, in our study, a 15-min PKC stimulation with 600 ng/mL PMA at 37 °C increased the number of apoptotic neutrophils threefold through phosphatidylserine detection, the first sign of early apoptosis. Lundqvist-Gustafsson et al. obtained similar results and observed an increase in the percentage of apoptotic neutrophils after a 30-min incubation with 0.05 µmol/L (equipotent to 30 ng/mL) PMA. Rapid PMA-induced neutrophil apoptosis observed in our study is likely due to respiratory burst and increased H_2_O_2_ production through intracellular NADPH oxidase (NOX), as Lundqvist-Gustafsson et al. demonstrated that PMA did not induce apoptosis in DMSO-differentiated HL-60 cells, which lack NADPH intracellular activity [37]. Suzuki et al. incubated porcine neutrophils with 100 ng/mL PMA for 5 h and reported decreased cell viability, which was temperature-dependent and increased with the incubation time at 37 °C. At low temperatures, neutrophils did not undergo spontaneous or PMA-induced apoptosis [38]. However, Okada et al. obtained different results and observed enhanced survival for eosinophils after PKC activation by 0.1 nmol/L (62 pg/mL) PMA [36]. The opposite effect may be due to different isoforms of activated PKC causing pro- or anti-apoptotic effects [35].

PMA is a well-known activator of NET formation in human neutrophils. This process requires activation of the Raf MEK–ERK pathway and depends on ROS produced by NOX. Increased intracellular calcium and ROS levels result in arginine deiminase 4 (PAD4) translocation from the cytoplasm to the nucleus. PAD4 converts arginine to citrulline in histones and weakens the DNA–protein bond [12]. Citrullination of histones induced by PAD4 is essential for NET formation and serves as a marker in NET identification [34]. Our study reported only a twofold increase in NETotic neutrophils after 600 ng/mL PMA stimulation for 15 min compared to a threefold increase in apoptosis. According to Takei et al., NETosis without signs of apoptosis occurred following 3–6 h of PMA incubation (30 ng/mL) [39]. In our study, we have seen the early stages of NETosis with the characteristic high fluorescence of CitH3 [34]. It is documented that suicidal NETosis is slow and requires more time than neutrophil apoptosis [39]. Therefore, we chose a short time of PMA incubation due to the 2-h pre-incubation with LAs. However, increased apoptosis after PMA stimulation observed in the study may be a limiting factor for neutrophil NETosis [40]. The longer incubation times with lower PMA concentrations would be better for investigating the complete NETosis process.

## Lidocaine and apoptosis

After 2 h of incubation, lidocaine induced early apoptosis in resting neutrophils at millimolar concentrations [41]. In contrast, PMA-stimulated neutrophils exposed to lidocaine showed no changes in the percentage of apoptotic cells at any of the drug concentrations analysed. Previous literature indicates that lidocaine activates apoptosis of human neutrophils only at high concentrations, which is clinically irrelevant. This pro-apoptotic effect is concentration-dependent and suggests a linear response.

Previous studies have indicated that 0.4 mmol/L lidocaine induced neutrophil apoptosis after 2 h of incubation by inhibiting ATP production and altering mitochondrial membrane potential. However, the analysis was performed on the in vitro model of resting neutrophils and did not reflect clinical settings when neutrophils are usually immunologically active [26]. The immunomodulatory effect of lidocaine, caused by increased apoptosis in the peripheral blood mononuclear cells (PBMC) pool after epidural anaesthesia, was reported in dogs but had little impact on the total lymphocyte number [31]. In concentrations observed during LA (1–10 µmol/L), lidocaine enhanced fibroblast apoptosis by increasing cAMP accumulation, inhibiting PKC and activating caspase-3. The pro-apoptotic effect of lidocaine in renal cells was mediated through decreased kinase B (Akt) and ERK signalling pathways [42]. Furthermore, lidocaine downregulated anti-apoptotic proteins, namely, Bcl-2 and Bcl-xl, and upregulated pro-apoptotic Bak and Bax [41]. We did not observe increased apoptosis under lidocaine doses of 2 and 20 µmol/L. However, low concentrations may have required a prolonged incubation, e.g., 24 h [26]. Our study reported no effect of lidocaine in PMA-stimulated granulocytes at any of the concentrations tested. Okada et al. demonstrated similar results when lidocaine of 1 mmol/L did not affect the viability of PMA-stimulated eosinophils [36]. PMA directly activates PKC and likely overcomes lidocaine’s protein Gq-mediated PKC inhibitory effect [36, 43, 44]. In neutrophils, lidocaine at 4 mmol/L reversed TNF-a-induced apoptosis after 12 h incubation [45], and together, these findings suggest lidocaine can inhibit or enhance apoptosis depending on the stimulation and type of cell [36].

## Ropivacaine and apoptosis

Ropivacaine is a relatively safe amide-type anaesthetic, preferentially used in perioperative settings. It modulates many leukocyte functions but to a lesser extent than lidocaine. After 2 h of incubation, ropivacaine induced early apoptosis in resting neutrophils at high concentrations. However, low concentrations showed anti-apoptotic effects by reducing the number of late-apoptotic neutrophils and increasing viability. Moreover, in PMA-stimulated cells, ropivacaine did not cause granulocyte apoptosis or viability changes. The biological response to low-dose ropivacaine was different from the higher dose, thus suggesting the drug’s non-linear, U-shape dosage effect. At low concentrations, ropivacaine may improve viability, although at high concentrations, it exerts apoptosis. This is the first report of the low-dose anti-apoptotic effect of ropivacaine related to human neutrophils and is of significant concern due to its common use in perioperative settings. Ropivacaine at low concentrations increases NO production in human neutrophils by enhancing iNOS expression [28]. The relationship between NO production and apoptosis is well-documented [46]. Low concentrations of NO may inhibit apoptosis through the decreased release of mitochondrial pro-apoptotic proteins, scavenging ROS or inducing the expression of cyclo-oxygenase-2, heme oxygenase-1 or metallothionein [46]. According to results from MTT assays, ropivacaine caused a cytotoxic effect on human neutrophils at 2 and 4 mmol/L after 2 h of incubation. Our results show that incubation of 2 h with 1.4 mmol/L ropivacaine increased the percentage of early apoptotic neutrophils with no changes in viability. A previous study by Kawasaki et al. reported no effect of ropivacaine on the apoptosis of human neutrophils. Decreased viability and increased late apoptosis were recently reported in HUVEC cells incubated with 367 µmol/L ropivacaine for 24 h. Interestingly, induced apoptosis involves activation of the intrinsic apoptosis pathway and LDH release [32]. Ropivacaine inhibited ERK1/2 phosphorylation, facilitated mitochondrial DNA (mtDNA) release, ROS formation and decreased levels of antioxidants, i.e., SOD1, SOD2, SOD3, TXN [32]. This may suggest that ropivacaine causes apoptosis due mainly to oxidative stress.

## Local anaesthetics and NETosis

In our study, lidocaine and ropivacaine did not affect the NETosis of resting neutrophils at any of the concentrations tested. In PMA-stimulated cells, there was a slight increase in the percentage of NETotic neutrophils under a dose of 4 mmol/L lidocaine (vs control and compared to the PMA-stimulated lowest concentrations), while ropivacaine had no effect, but we observed lower NETosis in resting neutrophils pre-treated with clinically relevant concentrations of the drug compared to PMA-stimulated control and the highest, 1.4 mmol/L dose of the drug. To date, there is a lack of literature regarding the mechanisms of NETosis modulation by LA. Experiments by Carmon-Rivera et al. investigated the activation of human neutrophil NETosis by levamisole, a drug with an affinity for the parasympathetic nervous system. Levamisole increased NET production by activating NOX and PAD4, leading to Akt phosphorylation, which acts as a molecular switch to guide cell death from apoptosis to NETosis. Moreover, previous investigations suggest that lidocaine decreases NET formation. Galos et al. reported decreased markers of NETs in the serum of patients following breast cancer surgery when intravenous lidocaine was used. Furthermore, reduced NETosis may be associated with less cancer recurrence. Kolle et al. reported increased time for maximal NETosis in neutrophils incubated with low-dose lidocaine and reduced incubation time [15]. The mechanism of lidocaine’s inhibitory effect on NET formation is not yet understood, although it is known that lidocaine inhibits high mobility group box-1 (HMGB-1) and granulocyte colony-stimulating factor (G-CSF), which may be necessary to induce NETosis. Intravenous infusions of lidocaine may attenuate the excessive immune response in the body following SARS-CoV-2 infection [11]. Our study reveals no changes to NETosis regarding the LAs type and concentrations; the observed increase is from PMA stimulation rather than the drug action. However, due to the paucity of evidence, the effects of LAs on neutrophil NETosis still need to be confirmed.

## Study limitations

Our study has some limitations. First, in vitro studies do not directly reflect the clinical conditions and the actual effects of the agents on the tested cells are not yet known. However, the experiments provide insight into the impact of LA at low concentrations, allowing the observation of potentially clinically significant outcomes. PMA stimulation was carried out for 15 min and caused fast phosphatidylserine exposure rather than NETosis. Apoptosis is a limiting factor in NET formation, and we did not separate NETotic neutrophils from those undergoing apoptosis. Furthermore, PMA stimulation of neutrophils is not a physiological model of neutrophil activation and thus may not be clinically significant. While lidocaine and ropivacaine exerted a different effect on resting and stimulated neutrophils, we can assume that they may also alter NETosis and apoptosis in primed or activated neutrophils that can be observed in nature. We did not conduct the incubation with LAs for more than 2 h, so we do not know how the time may change lidocaine and ropivacaine reactivity. The experiments should be repeated on active neutrophils from patients undergoing regional anaesthesia.

## Conclusions

This study found that lidocaine and ropivacaine may change apoptosis and NETosis of human neutrophils at low, clinically relevant concentrations. The effect depends on neutrophil stimulation and can differ between resting and active neutrophils. Ropivacaine tends to prolong the life of neutrophils at low concentrations, similar to that observed in plasma under LA. There were no differences between lidocaine and ropivacaine regarding NETosis and neither drug-induced NETosis in resting neutrophils. However, lidocaine slightly increased the number of NETotic PMA-stimulated neutrophils at high millimolar concentrations. Both drugs may change the two types of neutrophil death and thus may change the course of inflammation in perioperative settings, but are rather safe at low, clinically relevant concentrations found in plasma during local anaesthesia.

### Supplementary Information

Below is the link to the electronic supplementary material.

Supplementary file1 (DOCX 22 KB)

## Data Availability

The datasets used and analyzed during the current study are available from the corresponding author on reasonable request.
