# Supplementary material for: The effect of local anaesthetics on apoptosis and NETosis of human neutrophils in vitro: comparison between lidocaine and ropivacaine
Source: Hum Cell. 2023 Aug 17;36(6):2027–39. doi: 10.1007/s13577-023-00963-x (PMC10587218; doi:10.1007/s13577-023-00963-x)
Supplement: Supplementary file 1 — Supplementary file1 (DOCX 22 KB) [file 13577_2023_963_MOESM1_ESM.docx]

**The effect of local anaesthetics on apoptosis and NETosis of human neutrophils *in vitro* – comparison between lidocaine and ropivacaine**

Karolina Iwona Kulińska^1,*^, ORCID ID: 0000-0003-3192-8859, Sandra Szałkowska^1^, Mirosław Andrusiewicz^1^, ORCID ID: 0000-0002-8781-3447, Małgorzata Kotwicka^1^, ORCID ID: 0000-0002-9802-374X, Hanna Billert^2^, ORCID ID: 0000-0002-1698-8450

^1^ Chair and Department of Cell Biology, Poznan University of Medical Sciences, Rokietnicka 5D, 60-806 Poznan, Poland;

^2^ Chair of Anaesthesiology and Intensive Therapy, Poznan University of Medical Sciences, Przybyszewskiego 49, 60-355 Poznan, Poland

*corresponding author: [kulinska@ump.edu.pl](mailto:kulinska@ump.edu.pl) (K.I.K.)

Tab. S1. Effect of lidocaine and ropivacaine pre-incubation on apoptosis of resting and PMA-stimulated human neutrophils (n=8) Data are median with interquartile range in parentheses.

|  |  | **control (mmol/L)** | **lidocaine (mmol/L)** | | | **ropivacaine (mmol/L)** | | |
| --- | --- | --- | --- | --- | --- | --- | --- | --- |
|  |  | **0** | **0.002** | **0.02** | **4** | **0.0007** | **0.007** | **1.4** |
| unstimulated | Annexin V-/PI- | 92.0 (89.0-93.0) | 93.0 (93.0-94.0) | 93.0 (92.0-94.0) | 81.0 (74.0-92.0) | 95.0 (94.0-96.0)* | 94.0 (93.0-96.0) | 88.0 (78.0-94.0) |
|  | Annexin V+/PI- | 3.2 (1.4-3.3) | 3.1 (1.3-4.3) | 2.8 (1.4-4.1) | 10.0 (4.5-17.0)** | 3.0 (1.1-3.9) | 3.2 (2.4-4.5) | 6.9 (4.3-16.9) ** |
|  | Annexin V+/PI+ | 3.8 (2.7-6.9) | 2.7 (2.2-3.1) | 2.7 (1.6-3.3) | 4.8 (1.9-10.0) | 1.8 (1.3-1.8)** | 2.0 (1.2-2.2)** | 2.7 (1.9-4.5) |
|  | Annexin V-/PI+ | 1.5 (0.5-2.6) | 0.7 (0.2-2.2) | 0.8 (0.5-1.7) | 0.8 (0.3-1.9) | 0.7 (0.2-2.9) | 0.4 (0.2-1.1) | 0.4 (0.2-0.7) |
| PMA-stimulated | Annexin V-/PI- | 80.0 (68.0-88.0) | 85.0 (68.0-93.0) | 84.0 (75.0-92.0) | 83.0 (63.0-91.0) | 77.0 (70.0-87.0) | 74.0 (71.0-83.0) | 83.0 (79.0-90.0) |
|  | Annexin V+/PI- | 18 (9.2-29.0) | 13 (4.7-29.0) | 10 (6.2-21.0) | 16 (6.4-30.0) | 20.0 (8.0-24.0) | 21.0 (12.0-24.0) | 11.0 (7.1-20.0) |
|  | Annexin V+/PI+ | 2.4 (1.7-3.3) | 2.6 (0.9-3.6) | 3.2 (1.3-4.0) | 1.9 (1.2-4.7) | 3.8 (2.9-5.3) | 4.1 (2.6-6.7) | 2.3 (1.4-4.4) |
|  | Annexin V-/PI+ | 0.2 (0.1-0.8) | 0.3 (0.1-0.3) | 0.2 (0.1-0.6) | 0.1 (0.0-0.1) | 0.2 (0.1-0.3) | 0.2 (0.1-0.3) | 0.2 (0.0-0.4) |

Data are the median with interquartile range in parentheses; % apoptotic neutrophils are the sum of early and late apoptotic cells; ** *p* < 0,01, **p*<0.05 vs control; PMA – phorbol 12-myristate 13-acetate; Friedman with *post hoc* Dunn’s test

Tab. S2. The Mann-Whitney *U* test results for apoptosis and NETosis of resting and PMA-stimulated human neutrophils pre-exposed to lidocaine and ropivacaine.

|  | LA (mmol/L) | unstimulated | | | PMA-stimulated | | |
| --- | --- | --- | --- | --- | --- | --- | --- |
|  |  | NETosis [%] | Apoptosis [%] | *p*-value | NETosis [%] | Apoptosis [%] | *p*-value |
| control | 0 | 2.0 (0.34-2.9) | 5.8 (4.6-7.6) | **<0.0001** | 4.0 (1.5-5.8) | 17.0 (9.3-23.0) | **0.0002** |
| lidocaine | 0.002 | 0.7 (0.3-1.6) | 5.7 (4.8-7.0) | **0.03** | 2.9 (1.0-4.0) | 15.0 (6.5-31.0) | **0.01** |
|  | 0.02 | 0.6 (0.3-1.7) | 5.2 (3.6-6.2) | **0.01** | 2.0 (0.7-3.9) | 16.0 (7.8-24.0) | **0.01** |
|  | 4 | 1.7 (0.7-4.3) | 18.0 (7.4-25.0) | **0.0006** | 6.4 (1.4-11.0) | 17.0 (8.9-35.0) | **0.02** |
| ropivacaine | 0.0007 | 0.6 (0.2-1.1) | 5.3 (2.8-5.8) | **0.0002** | 2.2 (0.7-6.1) | 22.0 (13.0-30.0) | **0.0002** |
|  | 0.007 | 0.6 (0.2-0.8) | 5.2 (3.3-6.5) | **<0.0001** | 1.7 (1.3-5.5) | 25.0 (17.0-29.0) | **0.0002** |
|  | 1.4 | 1.2 (0.6-1.6) | 11.0 (6.1-20.0) | **<0.0001** | 4.3 (2.5-8.6) | 17.0 (9.6-21.0) | **0.003** |

Data are the median with interquartile range in parentheses; % apoptotic neutrophils are the sum of early and late apoptotic cells; PMA – phorbol 12-myristate 13-acetate; The Mann-Whitney *U* test
